# An Expert System for Quantification of Bradykinesia Based on Wearable Inertial Sensors

**DOI:** 10.3390/s19112644

**Published:** 2019-06-11

**Authors:** Vladislava Bobić, Milica Djurić-Jovičić, Nataša Dragašević, Mirjana B. Popović, Vladimir S. Kostić, Goran Kvaščev

**Affiliations:** 1 University of Belgrade-School of Electrical Engineering, 11000 Belgrade, Serbia; mpo@etf.rs (M.B.P.); kvascev@etf.rs (G.K.); 2 Innovation Center, School of Electrical Engineering, University of Belgrade, 11000 Belgrade, Serbia; milica.djuric@etf.rs; 3 Clinic of Neurology, School of Medicine, University of Belgrade, 11000 Belgrade, Serbia; ntdragasevic@gmail.com (N.D.); vladimir.s.kostic@gmail.com (V.S.K.); 4 Institute for Medical Research, University of Belgrade, 11000 Belgrade, Serbia

**Keywords:** decision support system, wearable inertial sensors, finger-tapping, automatic scoring, Parkinson’s disease, atypical parkinsonism, UPDRS

## Abstract

Wearable sensors and advanced algorithms can provide significant decision support for clinical practice. Currently, the motor symptoms of patients with neurological disorders are often visually observed and evaluated, which may result in rough and subjective quantification. Using small inertial wearable sensors, fine repetitive and clinically important movements can be captured and objectively evaluated. In this paper, a new methodology is designed for objective evaluation and automatic scoring of bradykinesia in repetitive finger-tapping movements for patients with idiopathic Parkinson’s disease and atypical parkinsonism. The methodology comprises several simple and repeatable signal-processing techniques that are applied for the extraction of important movement features. The decision support system consists of simple rules designed to match universally defined criteria that are evaluated in clinical practice. The accuracy of the system is calculated based on the reference scores provided by two neurologists. The proposed expert system achieved an accuracy of 88.16% for files on which neurologists agreed with their scores. The introduced system is simple, repeatable, easy to implement, and can provide good assistance in clinical practice, providing a detailed analysis of finger-tapping performance and decision support for symptom evaluation.

## 1. Introduction

Wearable sensors and advanced algorithms are increasingly being used for the development of new clinical support systems for more efficient diagnostics and the evaluation of symptom severity and disease progress in Parkinson’s disease (PD) [1]. This covers a wide range of applications assessing different symptoms, such as tremor, hypokinesia, rigidity, and bradykinesia.

Bradykinesia is one of the main manifestations of PD. It is evidenced as slowness of body movements, especially in tasks that require fine motor control [2]. In clinical practice, bradykinesia (as well as other motor symptoms) are usually assessed using the Unified Parkinson’s Disease Rating Scale (UPDRS), in which the third part of the examination is dedicated to motor skill evaluation (UPDRS III) [3]. Bradykinesia is evaluated using repetitive hand and leg movements, such as finger-tapping, hand opening/closing, pronation/supination, and foot (or toe) tapping [2]. As a part of the examination, patients are requested to repeatedly perform specified movements, as fast and with the biggest amplitude as possible, during some short period of time, usually 10–15 s [4,5,6,7], or for some specified number of repetitions, e.g., 10 times [8,9,10]. These movements are evaluated based on specifically defined criteria, including speed, amplitude, amplitude decrement, and number of hesitations or freezes. The performance is rated with scores ranging from 0 to 4, in which the lowest values correspond to normal movements, and higher values are given for more severe bradykinesia expressed through significant amplitude losses, decreasing speed, or an increased number of hesitations/freezes. However, in clinical practice, this examination is usually performed visually, which may result in subjective evaluation and rough quantification. Since precise evaluation represents a very important part of the long-term monitoring of the disease’s progress and patients’ response to therapy, researchers have dedicated their effort and time to design new systems that can be used for the objective evaluation and automatic scoring of symptom severity.

In the literature, different approaches are presented for the objective evaluation and quantification of PD motor symptom severity, including bradykinesia. The introduced methodologies differ in terms of applied instrumentation, analysed movements, measurement protocols, the size and composition of patients’ groups, and implemented signal processing and learning techniques. Some studies implement RGB or infrared camera systems for measuring clinically important repetitive hand and leg movements [5,11,12]. Although such systems can provide high-precision measurements, they have some limitations. They are expensive and require dedicated space for recording (they are bulky), which significantly limits their applicability in clinical settings [13]. Due to these limitations, wearable systems, such as smartphones [14], magnetic sensors [13], and inertial measurement units (IMUs) [7,8,9,15,16,17,18,19,20], are increasingly being applied for bradykinesia assessment. IMUs are small, lightweight, easy to mount, and do not require dedicated space for recording, which makes them more suitable for fast and reliable everyday clinical applications.

In the literature, bradykinesia is assessed by analysing different repetitive movements, including finger-tapping [8,13,21], hand opening/closing [16,17], hand pronation/supination [9,10,22], and toe tapping [23], as well as by simultaneous analysis of different movements [12,20,24]. From finger-tapping (FT) accelerometer data, researchers extract different features, describing the frequency and biomechanical properties of the movements, and use them as input into the ordinal logistic regression model for prediction of UPDRS FT scores [8]. It is shown that scores can be predicted with high predictive power (the Goodman–Kruskal Gamma score is 0.961). Four parameters extracted from the FT gyro data were found to be statistically correlated with clinical scores (from r=0.73 to r=−0.80) [7]. A similar approach is applied to a repetitive hand opening/closing task [16]. Signals are acquired with small IMUs and described by the dominant grasping frequency and mean angle, and fitted with the clinical UPDRS scores using a regression model [16]. It is shown that the predicted scores are highly correlated with the clinical scores (the determination coefficient is $r^{2}=0.99$).

A methodology that combines principal component analysis and multiple linear regression is applied to quantify bradykinesia severity in FT movements [13]. The method is applied to features that are extracted from the data recorded using magnetic sensors. It is shown that this approach can provide scores with a mean square error of 0.45 compared to the reference UPDRS FT scores. Another study presented a new approach that uses a motion capture system and dynamical features rather than standard spectral features for automatic scoring of the FT performance [5]. The results show strong and significant correlations with clinical scores.

In order to describe bradykinesia in multi-joint upper limb movements, researchers have introduced new performance indexes that are correlated with UPDRS bradykinesia scores and implemented for differentiation between PD patients with and without bradykinesia [22]. A similar approach is designed for the evaluation of bradykinesia in walking and sit-to-stand tasks, in which novel performance indices are successfully used for differentiation between healthy subjects and PD patients, and ON and OFF states in patients [25]. A support vector machine (SVM) classifier applied to spectral and nonlinear features achieves high-accuracy results (accuracy, sensitivity, and specificity above 97%) for the prediction of UPDRS FT scores (0–3) [19]. However, the method is applied to gyro signals recorded from healthy subjects who mimick the impaired movements of PD patients. In another study, SVM was successfully applied (error below 5%) for estimation of the severity of several symptoms (bradykinesia, tremor, and dyskinesia) in 12 PD patients using the features extracted from accelerometer data describing multiple upper and lower extremity movements [20]. SVM was also applied for estimation of bradykinesia severity in a study comprising 78 PD patients and 18 healthy subjects, who were instructed to perform hand opening/closing for 10 s [4]. It was shown that SVM can predict clinical scores with an accuracy of 95.349%. Decision trees, applied to features extracted from inertial signals, were also used for prediction of UPDRS scores for a pronation/supination task, showing a mean agreement of 0.48 with clinical ratings [10].

Although supervised machine learning algorithms provide prediction of clinical scores with high accuracy, the applied models are trained on a smaller dataset with subjectively defined data labels, which may cause subjectivity in the results as well. Because of that, some researchers have introduced different approaches to this topic. Decision rules can be designed to match exactly the criteria of the decision-making process and instructions applied in clinical practice. Fuzzy rules were applied for prediction of clinical scores using inertial data recorded during foot tapping [23] and hand pronation/supination movements in [9]. Their designed rules provide good results, with an accuracy of about 90%. Great Lake Technologies proposed a commercialized smartphone application, called Kinesia One, that provides clinical scores and subscores for different criteria for several bradykinesia tasks using a inertial sensor positioned on the index finger [26].

In this paper, we propose a new decision support system for the provision of clinical scores based on the use of inertial data describing finger-tapping movements. The proposed system uses novel metric and decision rules that are especially designed to capture and evaluate the relevant characteristics of the finger-tapping movement. The system provides very good results for data obtained from patients with idiopathic Parkinson’s disease but also with atypical parkinsonism. The output of the system comprises the kinematic features describing the finger-tapping performance, a graphical presentation of the recorded data with marked irregularities, and important changes in the signal and bradykinesia severity scores.

## 2. Materials and Methods

### 2.1. Measurement System

**Figure 1:**
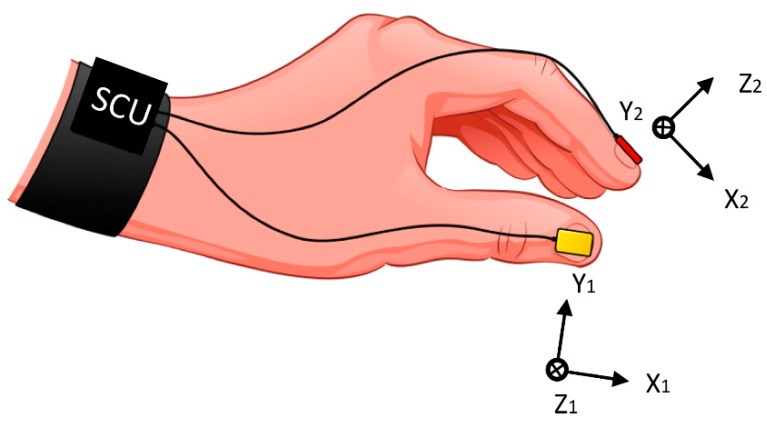
Illustration of the inertial sensor system, with local coordinate systems of the thumb $\left(X_{1},\;Y_{1},\;Z_{1}\right)$ and index finger $\left(X_{2},\;Y_{2},\;Z_{2}\right)$ sensors. SCU-sensor-control unit.

The used system comprises two miniature (10 × 12 mm) and lightweight inertial sensors with three-dimensional (3D) gyroscopes L3G4200 (STMicroelectronics, Geneva, Switzerland) positioned over the fingernails of the thumb and index finger, as shown in Figure 1 [6]. Inertial sensors are connected to sensor-control units (SCUs). An SCU acquires and wirelessly transmits sensor data to a remote computer, where custom-made software controls data acquisition (developed in CVI 9.0, NI LabWindows, National Instruments, Austin, Texas, USA).

### 2.2. Subjects

Fifty-six subjects were recruited for this study from the Clinic of Neurology, Clinical Centre of Serbia, Belgrade. The subjects included 13 patients (Gender: seven male/six female, Age: 62.23 ± 10.79 years) with idiopathic Parkinson’s disease (PD), 17 patients (Gender: five male/12 female, Age: 58.41 ± 6.41 years) with atypical parkinsonism multiple system atrophy (MSA), 14 patients (Gender: 11 male/three female, Age: 65.71 ± 9.33 years) with atypical parkinsonism progressive supranuclear palsy (PSP), and 12 healthy controls (HC) (Gender: four male/eight female, Age: 58.40 ± 7.78 years). The patients were tested during their “off” phase (after at least 12 h of treatment withdrawal, if possible). Descriptive statistics (average ± standard deviation and median) of the clinical data for each group of subjects are presented in Table 1, including the Hoehn and Yahr (H&Y) scale, total UPDRS, UPDRS-III (complete Motor examination scores), and scores given solely for the finger-tapping task by two neurologists, separately for the less- and more-affected hand.

### 2.3. Measurement Methodology

During the recordings, the subjects were sitting in the chair with their arms bent and supported at the elbow and hands placed in front of them. They were instructed to perform the finger-tapping test by tapping their thumb and index finger as quickly and as widely as possible for 15 s. Although instructions provided in the UPDRS test state that patients should tap their fingers 10 times [3], in this study, longer recordings were acquired to ensure that sufficient data for analysis were available.

In order to become accustomed to the instrumentation and measurement methodology, for each subject, several trials were recorded per hand, with one minute of rest between the trials. Each trial was also recorded with a video camera, which filmed the hand in a close-up view. The most representative recording (one for each hand) was used for further analysis. The recording was selected by the neurologists as the recording that fullfills the requirements of tapping duration and patients’ understanding of the given instructions. The testing of each subject was performed during one day at the Clinic of Neurology, Clinical Centre of Serbia, Belgrade. The examination was carried out in accordance with the ethical standards of the Declaration of Helsinki, and approved by the Ethical Committee of the School of Medicine, University of Belgrade. All of the participants provided informed consent prior to participation in the study.

### 2.4. Scoring by Neurologists

The recorded video data were later examined and scored by two neurologists with more than 10 years of experience, based on their knowledge and experience and the instructions given in the UPDRS, Part III–Motor examination, task 3.4 Finger tapping. The neurologists were blinded to the subjects’ identity, since the video data show a close-up view of each subject’s hand. The scores were given for each patient, separately for the left and right hand. The scores given by neurologists are provided in Table 1, in the last two columns. The scores given for the patients were provided separately for the less- and more-affected hand (averaged for all patients per group), whereas, in the case of healthy controls, the scores were averaged for both hands and all HC participants.

### 2.5. Data Processing and Analysis

**Figure 2:**
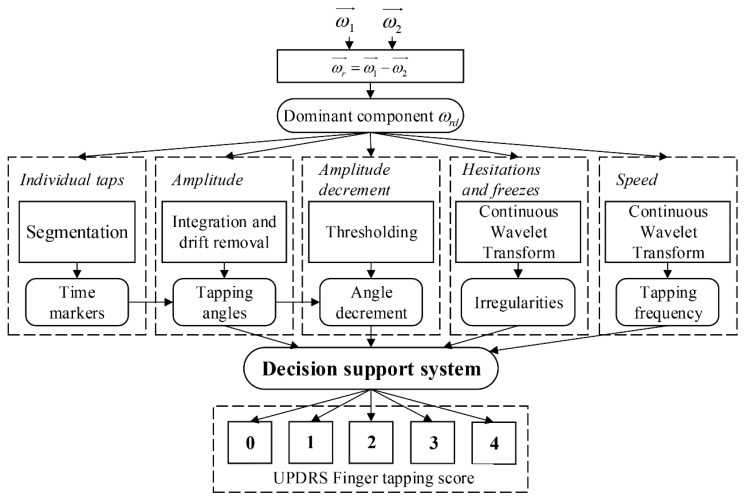
Block diagram of the expert system for UPDRS finger-tapping score calculation.

The gyro data were recorded with a sampling frequency $f_{s}=200\;\mathrm{Hz}.$ Calibrated data were processed in Matlab 9.0 R2016a (MathWorks, Natick, MA, USA). The flowchart of the expert system for calculation of UPDRS finger-tapping scores is presented in Figure 2. The inputs to the expert system are angular velocities from the thumb ($\vec{\omega_{1}}$) and index finger sensors ($\vec{\omega_{2}}$). No pre-processing was performed on the input signals. Upon the sensor’s placement, the coordinate system of the thumb $\left(X_{1},\;Y_{1},\;Z_{1}\right)$ and the coordinate system of the index finger $\left(X_{2},\;Y_{2},\;Z_{2}\right)$ were rotated with respect to each other (Figure 1). The angular velocities were transformed and analyzed from the index-finger coordinate system. The relative angular velocity of the thumb with respect to the index finger was calculated $\vec{\omega_{r}}=\vec{\omega_{1}\;}-\vec{\omega_{2}}$ [27]. The dominant component of the relative angular velocity $\omega_{\mathrm{rd}}$ was automatically selected and used as the input in further data processing and analysis [27]. It was shown that, in most cases, the dominant component of the relative angular velocity is about the $Y_{2}-$axis of the index-finger coordinate system. In other cases (when this component is not dominant), the coordinate system of the index finger was rotated, so the new $Y_{2}-$axis represents the dominant rotation.

Further analysis was divided into one pre-processing block for segmentation to individual taps and four blocks that calculate features to describe criteria defined in the UPDRS test: tapping amplitude, amplitude decrement, hesitations and freezes, and tapping speed. The calculated features are then used as the input to the decision-support system. As the result, a complete analysis of the patient’s finger-tapping performance, including the finger-tapping score (0–4), is provided.

#### 2.5.1. Individual Taps

**Figure 3:**
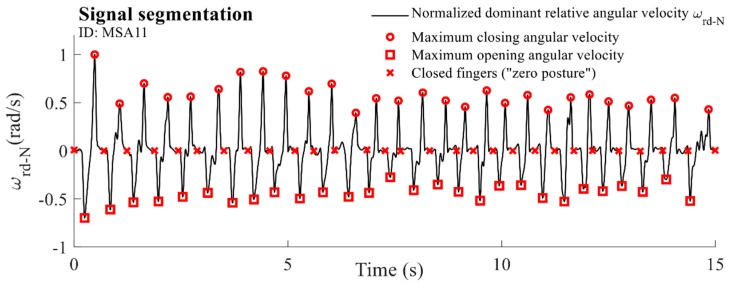
An example of the normalized dominant component of the relative angular velocity $\omega_{\mathrm{rd}}$ for one MSA patient (ID: MSA11) with extracted markers.

In order to evaluate characteristics of the tapping performance for individual taps, segmentation of the dominant component of the relative angular velocity $\omega_{\mathrm{rd}}$ was performed. A moving-average filter was applied to the observed signal with a span equal to $(f_{S}/f_{0})/2$, where $f_{0}$ represents the basic tapping frequency extracted from the spectrum. The filtered signal was normalized to its maximum value. From the obtained sequence, areas above 0.1  and below −0.1  were identified, corresponding to regions where positive peaks and negative valleys are located, respectively. Local extrema were identified for each of the extracted regions. Positive peaks correspond to the maximal closing velocity (circles, Figure 3), whereas negative valleys represent moments when fingers achieve the maximal opening velocity (squares, Figure 3). The samples in which the smoothed angular velocity $\omega_{\mathrm{rd}}\;$ passes through a zero value for the first time were identified between each neighbouring maximum and minimum marker. These samples represent the moments when fingers are closed (“zero posture”). The sequence was complemented with the first and last sample. The finally obtained samples were identified as time markers for drift removal and segmentation on individual taps (crosses, Figure 3).

#### 2.5.2. Amplitude

**Figure 4:**
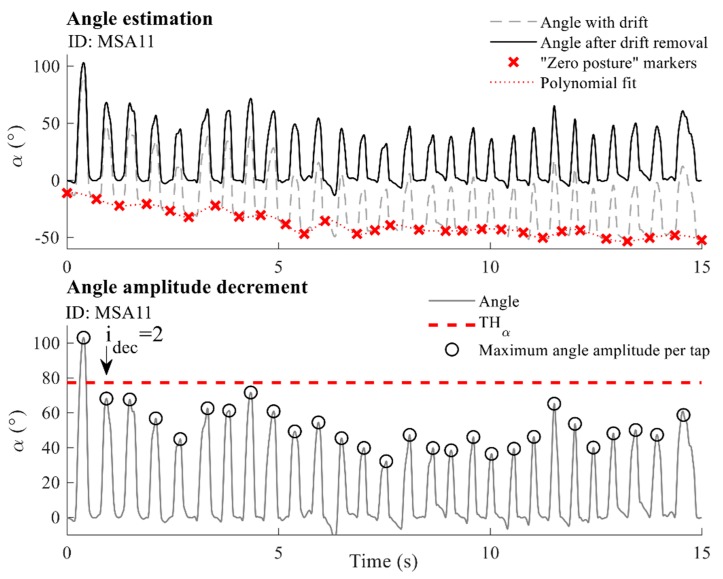
Upper panel: Angle estimation. The dashed grey line marks the drifted angle sequence, and the solid black line corresponds to the angle sequence after drift removal. Red crosses show “zero posture” markers, and the dotted red line presents the polynomial fit used for drift removal. Lower panel: Angle amplitude decrement. The solid grey line shows the angle sequence, whereas black circles mark the angle amplitudes (highest finger apertures) per tap. The dashed red line presents the threshold $TH_{\alpha }$ used for the detection of decreased amplitudes. The example is given for one MSA patient (ID: MSA11).

One of the evaluation criteria is the tapping amplitude, which evaluates how widely subjects can tap their fingers. The finger-tapping amplitude is defined as the angle that fingers formed during repetitive tapping movements. The tapping angle was calculated by integrating the dominant component of the relative angular velocity $\omega_{\mathrm{rd}}$ [27]. The drift was removed by using a third-order polynomial fitted (approximation) through markers corresponding to moments where fingers are closed (i.e., the angle is equal to zero, red crosses in Figure 4). Upon the drift’s removal, the obtained angle sequence was segmented into individual taps using the same time markers. The highest aperture of the fingers (the biggest angle that fingers form) was found for each individual tap and is expressed in degrees α(i) (°) (black circles in Figure 4, lower panel). The final parametric result was calculated as the average of the maximum angles calculated for each tap-$\alpha_{\mathrm{av}}$ (°).

#### 2.5.3. Amplitude Decrement

Physicians evaluate the amplitude decrement according to the part of the tapping sequence at which the amplitude starts to decrease. In order to objectively quantify changes of the tapping amplitude, we observed tap-to-tap changes in the highest finger apertures calculated for individual taps α(i). The angle amplitude of each individual tap was compared with the previously achieved maximum aperture of the fingers. The threshold $TH_{\alpha }=75\%$ of the value of the previous maximum finger aperture was selected as the optimum (as shown in Figure 4, lower panel). This threshold was heuristically determined through extensive analysis of the used signal database. Threshold values from 50% to 90% (with a step of 5%) were chosen and tested. The threshold of 75% provides the best results for the prediction of scores. It was shown that higher threshold values cause detection of very small amplitude changes, which can appear due to normal movement variability. Lower threshold values detect angle decrements later in the tapping sequence, with some delay compared to the first real significant decrement. Indices of all taps that satisfy this criterion for the amplitude decrease were extracted using the chosen threshold $TH_{\alpha }$ (for the example shown in the lower panel of Figure 4, all taps are below the threshold except for the first one, which is used as the reference for the calculation of the threshold). The indices of the first tap from the obtained sequence were selected as the final parametric result and marked with $i_{dec}$ (for the example in Figure 4, that is the second tap and, therefore, $i_{dec}=2$).

#### 2.5.4. Hesitations and Freezes

Hesitations and freezes are manifested as irregularities or breaks of the tapping rhythm that may occur in different moments of the tapping performance and represent an important part of the finger-tapping evaluation. The continuous wavelet transform (CWT) was applied for the detection and localization of disruptions of the tapping rhythmicity [28]. It is a time-frequency analysis method that is suitable for the analysis of transient changes and spikes in rhythmic behaviour [29].

**Figure 5:**
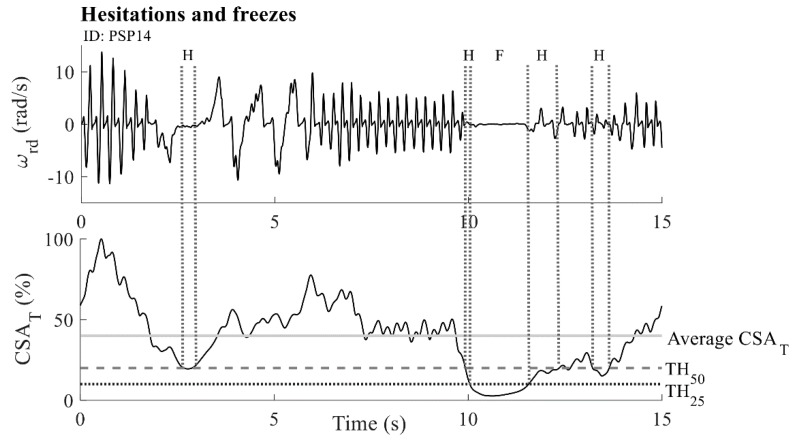
Calculation of hesitations and freezes: angular velocity $\omega_{\mathrm{rd}}$ (upper panel) and calculated $CSA_{T}$ characteristic (bottom panel). The solid grey horizontal line marks the average $CSA_{T}$ value. The dashed grey horizontal line corresponds to the upper threshold $TH_{50}=50\%$ of the $CSA_{T}$ average value. The dotted black horizontal line shows the lower threshold $TH_{25}=25\%$ of the $CSA_{T}$_-_ average value. Similarly, dotted grey vertical lines show areas that are classified as hesitations (an “H” mark) and freezes (an “F” mark). The example is given for one PSP patient (ID: PSP14).

CWT was applied on the dominant component of the relative angular velocity ($\omega_{\mathrm{rd}}$). The CWT method based on the Fast Fourier transform algorithm was used, together with the mother wavelet function from the complex Morlet family (center frequency *f*_0_ = 1 Hz and time-frequency resolution σ = 0.7). A matrix of complex CWT coefficients was obtained as a result. We introduced a cross-sectional area by summing the CWT coefficients perpendicular to the time axis. The obtained characteristic was normalized with respect to its maximum value and is expressed as a percentage ($CSA_{T}$ (%)). In this way, we obtain a characteristic that describes the temporal changes in the tapping activity [28]. An example of the $CSA_{T}$ characteristic for one patient is presented in Figure 5, lower panel. The samples were then divided according to two thresholds: $TH_{50}=50\%$ of the average $CSA_{T}$ value, and $TH_{25}=25\%$ of the average $CSA_{T}$ value (the dashed grey and dotted black vertical lines in Figure 5, respectively). Samples with values below $TH_{50}$ and above $TH_{25}$ threshold were considered to be parts of the hesitation sequences (Figure 5, dotted grey vertical lines, with an “H” mark), whereas samples with the smallest amplitude (below $TH_{25}$) were considered to be parts of freezes (Figure 5, dotted grey vertical lines, with an “F” mark). If a hesitation sequence lasts three times longer than the subjects’ average tapping frequency, then it is considered to be a freeze sequence. In addition, very short sequences (shorter than one half of the subjects’ average tapping frequency) were discarded from the analysis. The parametric result comprises the number of hesitation sequences $H_{num}$ and the number of freeze sequences $F_{num}$. Using the average $CSA_{T}$ value for thresholds ensures that the detection of irregularities is adapted to the intrinsic properties of each signal, considering the signal parts with significant losses in power (below 50% and 25% of the average) as irregularities. The values of the applied thresholds were verified through an extensive search of the database. All detected irregularities were confirmed by neurologists during their visual inspection of video recordings.

#### 2.5.5. Speed

**Figure 6:**
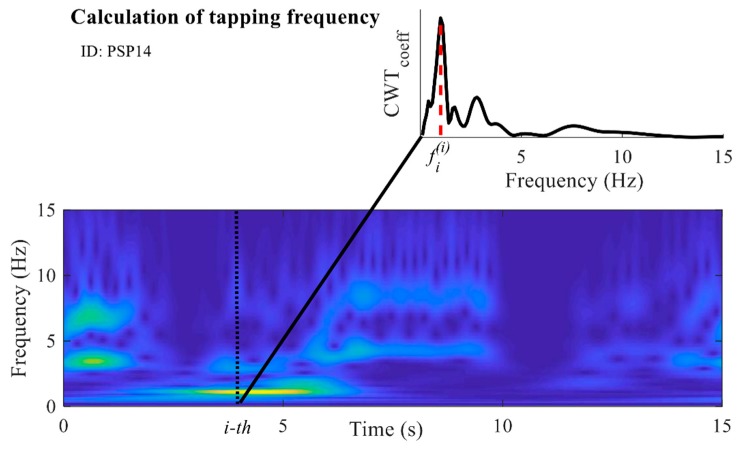
Calculation of the frequency characteristic: scalogram of the obtained continuous wavelet transform (CWT) coefficients. The dashed black line marks the *i*-th sample. The CWT coefficients at the *i*-th sample are presented in the smaller upper panel. The red dashed line in the upper panel marks the frequency with the highest amplitude of the CWT coefficients for the *i*-th sample (referred to as ${f_{i}}^{\left(i\right)}$). The example is given for one PSP patient (ID: PSP14).

An important criterion for evaluation of bradykinesia in the finger-tapping task is the tapping speed. During the evaluation, neurologists examine how fast subjects are tapping. If subjects tap faster, then during those 15 s of the tapping test they perform a larger number of taps, and vice versa. Although this can also be evaluated from the number of performed taps and their duration, by using the calculated matrix of CWT coefficients, the dominant tapping frequency can be found for each time sample. In this way, all changes of the tapping rhythm are assessed, detected, and included in the analysis. The vector of coefficients corresponding to one sample was extracted from the CWT matrix. From the obtained vector, the most prominent frequency was calculated as the frequency at which the coefficient with the highest value is located (as shown for the *i*-th sample in Figure 6). The procedure was repeated for all samples. In this way, the new frequency characteristic $f^{\left(i\right)}$ was obtained. The average value of the frequency characteristic $f^{\left(i\right)}$ was calculated and is marked as $f_{av}^{\left(i\right)}$ (Hz).

#### 2.5.6. Decision Support System

In the UPDRS motor scale, Part III–Motor examination, task 3.4 Finger tapping [3], instructions for bradykinesia evaluation are given as follows:

0 **Normal:** Regular rhythm, without hesitations or freezes. Fast movement, large amplitude, no amplitude decrement.
1 **Slight:** Any of the following: (a) the regular rhythm is broken with one or two interruptions or hesitations of the tapping movement; (b) slight slowing; (c) the amplitude decrements near the end of the 10 taps.
2 **Mild:** Any of the following: (a) three to five interruptions during tapping; (b) mild slowing; (c) the amplitude decrements midway in the 10-tap sequence.
3 **Moderate:** Any of the following: (a) over five interruptions during tapping or at least one freeze in ongoing movement; (b) moderate slowing; (c) the amplitude decrements starting after the first tap.
4 **Severe:** Cannot or can only barely perform the task due to slowing, interruptions, or decrements.

Each hand is evaluated separately, in terms of speed, amplitude, hesitation and freezes, and decrementing amplitude. These criteria are described with the introduced features, which are then fed to the decision support system. The input feature set includes the average tapping angle $\alpha_{av}$, the average frequency $f_{av}^{\left(i\right)}$, the index of the first tap with a significant angle amplitude decrement $\;i_{dec}$, the number of hesitations $H_{num}$, and the number of freezes $F_{num}$. The rules are defined separately for each feature to give the subscores for each criterion, which are afterwards used for the calculation of the final score.

As indicated, the lowest score corresponds to “normal” movements. Therefore, the first step is to find values that could be considered to be the reference for normal movements. The defined methodology was initially applied to a signal database from the control group that included a subset of healthy controls with no signs of bradykinesia (scored with 0). During the examination of the video files, it was noticed that both patients and healthy controls performed the tapping task in two different ways. In the first group, the subjects tapped as widely as possible at the highest speed that allows for such a tapping. In the other group, the subjects tapped with smaller amplitudes, but at their fastest pace. Using the parameters describing the tapping speed and amplitude ($\alpha_{av}$ and $f_{av}^{\left(i\right)}$, respectively), the selected healthy controls were divided into two clusters using the k-means algorithm. The coordinates of the cluster centers were used as the measure for discriminating the two types of tapping performance. From all three groups of patients, we randomly selected 50% percent of the files and assigned them to the testing group. By calculating the distance between the center of the clusters and the data ($\alpha_{av}$, $f_{av}^{\left(i\right)}$) obtained from the testing group, each patient was assigned to one of the two defined clusters ($C_{1}$, “wider and slower”; $C_{2}$, “narrower and faster”). The scores provided by the neurologists are given as the final score and do not provide information about different aspects (characteristics) of the performance that were analyzed. Because of that, it was necessary to apply an unsupervised learning algorithm to analyze properties of the features and find a natural grouping among the data. Testing data corresponding to one of the parameters $(\alpha_{av}$ and $f_{av}^{\left(i\right)})$ and one of the clusters ($C_{1}$ or $C_{2}$) were additionally divided into four clusters (corresponding to scores 0–3) using the k-means algorithm. Although there are five scores in the UPDRS test, the data were divided into four clusters, since the highest score (corresponding to the worst performance) is assigned to patients that barely perform the task (the movement is affected by multiple types of disturbances simultaneously). The coordinates of the cluster centers $\left(c_{1},\;c_{2},\;c_{3},\;c_{4}\right)$ were used for calculation of decision boundaries:

(1) $$b_{i}=\frac{c_{i}+c_{i+1,}}{2};i=1,2,3$$

where $c_{i}$ and $c_{i+1}\;$ represent the centers of two neighbouring clusters and $b_{i}$ represents the calculated boundary separating the two scores. The procedure was repeated for both clusters $C_{1}$ and $C_{2}$, and for both parameters $\alpha_{av}$ and $\;f_{av}^{\left(i\right)}$, separately (1: $C_{1}$ and $\alpha_{av}$, 2: $C_{2}$ and $\alpha_{av}$, 3: $C_{1}$ and $f_{av}^{\left(i\right)}$, 4: $C_{2}$ and $f_{av}^{\left(i\right)}),\;$ resulting in four sets of boundaries, each with three values. For each analyzed file, decision boundaries for the $\alpha_{av}$ and $f_{av}^{\left(i\right)}$ features were selected from those four sets. If this coordinate pair $(\alpha_{av}$, $f_{av}^{\left(i\right)})$ is closer to the center of the cluster $C_{1}$ than to the center of the cluster $C_{2}$, then the patient’s file was assigned to the cluster $C_{1}$ and decision boundaries ($b_{\alpha 1,2,3}$ and $b_{f1,2,3}$) for cluster $C_{1}$ were selected, and vice versa. Decision boundaries for the remaining features ($i_{dec}$ and $H_{num},\;F_{num}$) were set to match the instructions and criteria given within the UPDRS scale (as mentioned above).

**Figure 7:**
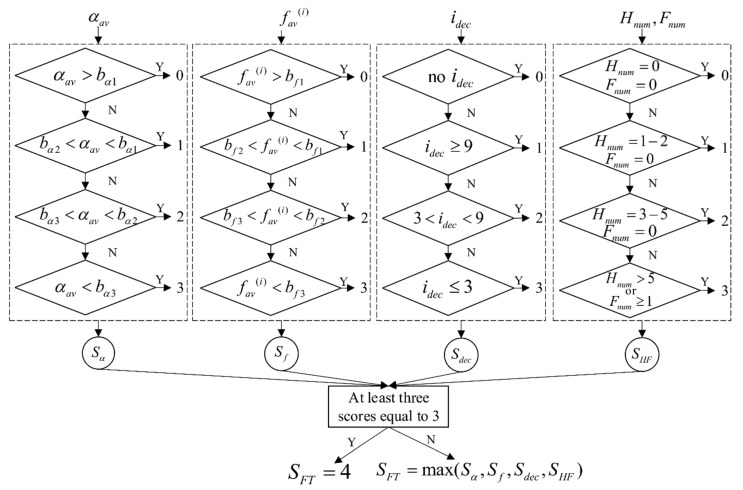
The block scheme of the decision support system. The system is divided into four processing blocks (bordered with dashed black rectangles). The inputs to the blocks are the calculated features: $\alpha_{av}$, $f_{av}^{\left(i\right)}\;i_{dec}$, and $H_{num},\;F_{num},$ respectively. Each block implements rules and assigns a subscore for the input feature. The final score $S_{FT}$ is decided based on the results obtained from all four blocks.

The block scheme of the decision support system is presented in Figure 7. The first part of the decision-making process is divided into four blocks (each bordered with dashed black line). The inputs of these blocks are the calculated features: $\alpha_{av}$, $\;f_{av}^{\left(i\right)},\;i_{dec}$, and $H_{num},\;F_{num}$, respectively. For each feature, a subscore is calculated separately, based on the range within which the feature value is located. In this way, the four processing blocks result in four subscores: $S_{\alpha },S_{f},S_{dec}$, and $S_{HF}$, respectively. If the subscore “3–Moderate” is obtained for at least three out of four features, then the final score $S_{FT}$ is set to be “4–Severe”. Otherwise, the final score $S_{FT}$ is selected as the maximum obtained subscore among the four subscores corresponding to the individual features.

#### 2.5.7. Statistical Analysis and Evaluation

To find the agreement between the scores obtained from two neurologists (raters), Cohen’s kappa statistics for finding intra-rater reliability among categorical data were applied. The results obtained from the decision support algorithm were compared with the scores given by the neurologists. The performance was measured using the confusion matrix and the accuracy of the proposed method, expressed as the percent of equally assigned scores. Initially, results were evaluated for all recordings (Case I), and later for the recordings equally scored by both raters (Case II).

## 3. Results

The intra-rater reliability was calculated with the Cohen’s Kappa statistic and it equals to κ = 0.79, showing some discrepancy between raters’ scores. This result is expected, since the scores are provided based on their visual and subjective estimation. Overall, 87 recordings obtained from 44 patients (PD: 26 recordings, MSA: 34, PSP: 27) were included in the analysis, as well as 24 recordings obtained from 12 healthy controls. The descriptive statistics for the introduced features are given in Table 2 for each group of subjects separately.

The highest values for the $\alpha_{av}$ and $f_{av}^{\left(i\right)}$ parameters were obtained for HC. Among patients, the PD group achieved the biggest angle amplitude values (on average); however, their tapping frequency was found to be lower (on average) compared to the PSP group. This discrepancy shows that we need to discriminate between the two types of movements and, consequently, use two sets of decision boundaries for these two features. The feature describing the angle decrement $\left(i_{dec}\right)$ was found to be comparable among patients. Although some HC also show a decrease in the angle amplitude, this is observed later in the tapping sequence (usually after the 10th tap). PD patients did not experience any freezing during the performance, whereas the number of hesitations was found to be comparable among groups. Among HC, none of the subjects experienced either hesitation or a freeze.

**Figure 8:**
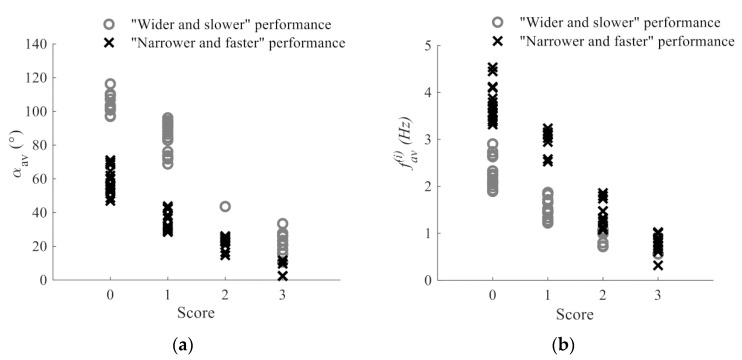
Dependency of: (**a**) calculated scores and the $\alpha_{av}$ feature; (**b**) calculated scores the and $f_{av}^{\left(i\right)}$ feature. Grey circles mark samples that are assigned to the $C_{1}$ cluster (“wider and slower” performance), whereas black crosses correspond to members of the cluster $C_{2}$ (“narrower and faster” performance).

Figure 8 shows the obtained angle $\alpha_{av}$ and frequency $f_{av}^{\left(i\right)}$ features versus the calculated scores. The performance clusters are shown using the color- and shape-coded representation. It can be seen that the $\alpha_{av}$ and $f_{av}^{\left(i\right)}$ features decrease with higher scores, which is in line with the criteria that is observed within the UPDRS. In addition, it can be confirmed that files assigned to the cluster $C_{1}$ are characterized by larger angle values and a lower tapping frequency, whereas the cluster $C_{2}$ includes files with a lower angle amplitude and a larger tapping frequency.

**Figure 9:**
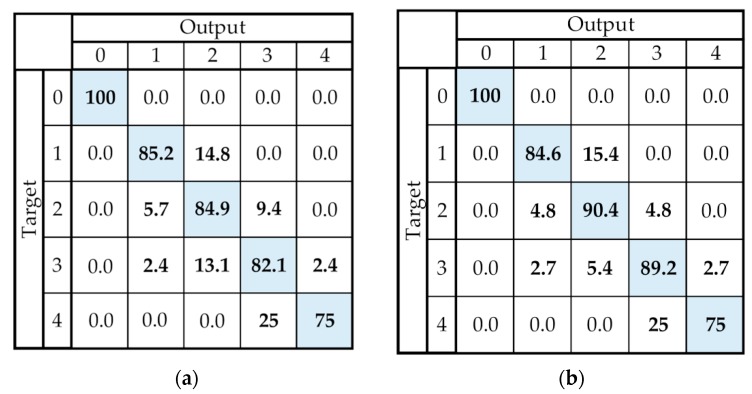
Presentation of results using the confusion matrix. (**a**) Case I—The result obtained when all recordings are included. (**b**) Case II—The result obtained using only recordings on which both raters agreed. The cells on the diagonal of the confusion matrix show the overall success rate for each score (expressed as a percentage (%)), whereas the cells outside the diagonal show the error rate for the scores (expressed as a percentage (%)).

The results of the expert system are presented in Table 3, for each group separately, as well as the summary for all patients. The results from the left column were obtained using all the recordings (Case I—87 recordings, PD: 26, MSA: 34, PSP: 27) and averaged for two raters, whereas the right column shows results obtained using only the recordings equally scored by both raters (Case II—76 recordings, PD: 25, MSA: 29, PSP: 22). Results are also presented in Figure 9 by a confusion matrix.

When all recordings are included in the analysis, comparable results are obtained for all three groups of patients. It is shown that the decision support system provides results that agree with the scores of the neurologists with a good accuracy (above 80%). This result is improved when only recordings equally scored by both raters are included in the analysis, achieving nearly 90% matching between the system results and neurologists’ estimates.

**Figure 10:**
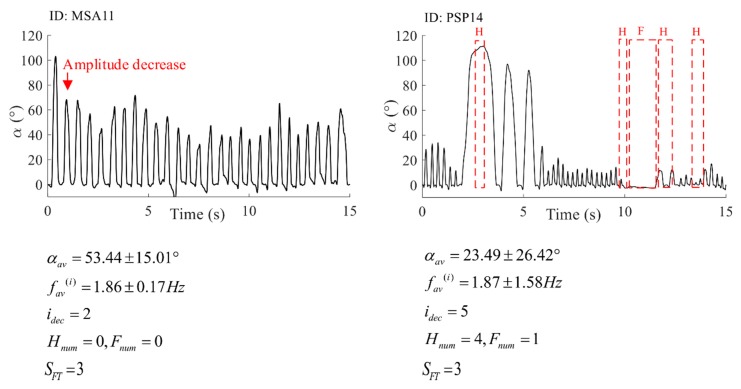
The result of the expert system comprising a graphical representation with detected irregularities, calculated features, and the final score. The example is given for one MSA patient (ID: MSA11), right hand, and one PSP patient (ID: PSP14), right hand.

The scores evaluated by the proposed decision system and the scores given by neurologists (Figure 9) do not exceed a one score difference, except for one patient. In Figure 10, we present the results of the expert system. The example is given for two patients who were equally scored by both raters and our expert system (score $S_{FT}=3$).

## 4. Discussion

In this paper, we introduced a new methodology that enables objective evaluation and quantification of the finger-tapping test that is usually used for bradykinesia assessment in patients with Parkinson’s disease. The system comprises two miniature and lightweight gyro sensors that record the motion of fingers. The methodology for signal quantification is based on the use of simple, automatized, and repeatable signal-processing techniques. In this study, 15 s long finger-tapping sequences were recorded and analyzed. However, the methodology is applicable to other approaches as well (e.g., a 10-tap-long sequence). Patients with finger-tapping bradykinesia severity ranging from 0 to 4 were included in this study. Most of the studies in the literature include severity stages up to 3, indicating that patients with the highest severity cannot perform specified tasks at all. In this study, we included three patients that barely managed to perform the finger-tapping task with one of their hands. However, their performance was poor and affected by multiple performance disturbances. Therefore, they were evaluated with the highest bradykinesia severity score (4). In this way, the performance of the proposed system was examined for the entire range of severity stages.

The parametric result comprises features that can be directly correlated with biomechanical properties of the movement and can, therefore, be used to assist physicians during the assessment of bradykinesia in repetitive finger-tapping. For such a purpose, we implemented the time-frequency method continuous wavelet transform, which allows us to evaluate temporal changes in the tapping frequency and to detect irregularities in rhythmic tapping behaviour, such as hesitation and freezes. Numerical integration and drift removal were used for estimation of tapping angle apertures that are calculated for each tap. Temporal changes in the angle apertures were found and described by the index of the first tap with a significant amplitude decrement (compared to previously achieved tapping angle amplitudes). The final feature set is used as the input into the decision support system.

Although the literature suggests that machine learning algorithms can predict scores with a high degree of accuracy, labels used for learning are given by physicians, which may cause subjectivity in the obtained results. Typically, only a few dozen recordings are used for learning, which is not a sufficient number of training examples to obtain a clinically acceptable system. Therefore, the decision support system consists of simple rules with decision boundaries designed to match the UPDRS scoring criteria. The decision boundaries for the tapping frequency and angle amplitude are defined according to the feature values obtained from the healthy controls and testing group of patients using the clustering techniques. As shown in Figure 8, smaller angle amplitudes and frequencies correspond to higher scores, which is in line with the criteria that are defined within the UPDRS. The boundaries differ, and they are selected based on the type of movement: wider and slower (cluster $C_{1}$, grey circles in Figure 8) or narrower and faster (cluster $C_{2}$, black crosses in Figure 8). In addition, using the clustering techniques, the boundaries are not defined linearly or empirically, but solely based on the grouping of some randomly selected testing data. The decision boundaries for two other features, i.e., the criteria, are defined to match the rules defined in the UPDRS.

The results of the decision support system (Table 3) demonstrate that the expert system has achieved an overall accuracy of 83.33 ± 6.50% (averaged for both raters), whereas this result is 82.69 ± 2.72% for PD, 82.36 ± 8.32% for MSA, and 83.76 ± 7.86 for PSP patients. By analyzing the recordings that were evaluated with the same score by both raters, the overall accuracy of the system is increased, achieving 88.16%, whereas this result is 84.00% for PD, 89.65% for MSA, and 90.91% for PSP patients. In the latter case, the decision support system provides wrong scores for only nine recordings (out of 76 recordings). The decision support system provides very good results, even for atypical parkinsonism, in which finger-tapping can be performed differently compared to the typical PD form [27]. By using a larger number of patients, fine tuning of the decision boundaries can be performed, providing even better results. The differences in Table 2 between the obtained parameters indicate that this methodology can be used as the basis for differential diagnostics of typical and atypical parkinsonism.

As shown in the confusion matrices in Figure 9, the quantification errors are equal to a one score difference, except for one PD patient, in which our system provides results that are two scores lower than the score from both raters. The question that arises is whether a scale with such a small resolution and only four grades of performance is sufficient. Figure 10 shows the results of the expert system for two patients evaluated by the same score ($S_{FT}=3)$. These two performances are different: the first one has a lower tapping frequency and higher angle apertures, with a significant decrease in the angle amplitude after the first tap. The second performance has large variations in frequency and angle amplitudes, as well as four hesitations and one freeze. For the first patient, the resulting score is given based on the early amplitude decrease, whereas, for the second patient, the score is given based on the number of performance irregularities. In the UPDRS instructions, it is said that a score is to be given if any of the criteria (speed, amplitude, amplitude decrease, hesitations/freezes) are satisfied. If different or more than one criterion is satisfied for different patients, they cannot be compared. Due to that fact, some researchers have introduced continuous scoring of repetitive hand motions that are used for bradykinesia evaluation. Although they provide a more detailed scoring system, this evaluation does not correspond to standardized clinical scores and may be confusing to physicians.

The proposed system provides a complete analysis of repetitive finger-tapping performance, objective measures of important biomechanical properties of the movement, and a graphical presentation of the recorded data with specific changes and irregularities marked in the data. The system can differentiate between different types of performances and provides decision support through automatically calculated scores and subscores for different criteria for the evaluation of movements. The scores are given using rules that are specifically designed to match the universal clinical criteria for evaluation of bradykinesia severity in repetitive finger-tapping movements. In addition, the system was tested on patients with different forms of parkinsonism and at different disease stages, and for the full range of symptom severity (from normal to severely impaired movements). The proposed expert system is detailed and objective, and, therefore, it can be used as a powerful support tool in clinical practice for the evaluation of symptom severity, monitoring the disease’s progress and a patient’s response to therapy, and comparisons with other patients. In the future, an intuitive graphical interface for a software application will be developed to provide a graphical presentation, numerical results for features, scores and subscores, and a statistical analysis.

Future work will also include collaborations with other researchers and groups to obtain larger databases and augment the data for analysis in terms of included subjects and the number of tests used to assess bradykinesia and other motor symptoms. We also plan to develop a metric to be used for more efficient differential diagnostics of typical and atypical parkinsonism.

## Figures and Tables

**Table 1:** Descriptive statistics of the subjects’ data.

Group	Statistics	H&Y	UPDRS Total	UPDRS III	FT_N1_ Score		FT_N2_ Score	
					Less AH	More AH	Less AH	More AH
PD	Avg ± std	1.80 ± 0.79	42.60 ± 16.93	24.60 ± 9.07	1.67 ± 0.89	2.17 ± 0.94	1.75 ± 0.97	2.17 ± 0.94
	Median	2	36	19.5	2	2	2	2
MSA	Avg ± std	3.18 ± 0.75	77.73 ± 13.70	46.64 ± 9.08	2.31 ± 0.70	2.81 ± 0.54	2.38 ± 0.72	2.81 ± 0.54
	Median	3	79	45	2	3	2.5	3
PSP	Avg ± std	3.45 ± 0.93	74.45 ± 20.08	42.91 ± 13.14	2.17 ± 0.94	2.62 ± 0.77	2.08 ± 0.79	2.77 ± 0.73
	Median	4	79	46	2.5	3	2	3
HC	Avg ± std	/	/	/	0.44 ± 0.63		0.50 ± 0.73	
	Median	/	/	/	0		0	

**^1^** PD—Parkinson’s disease; MSA—Multiple system atrophy; PSP—Progressive supranuclear palsy; HC—Healthy controls; H&Y—Hoehn and Yahr scale; UPDRS–Unified Parkinson’s Disease Rating Scale; UPDRS III—Unified Parkinson’s Disease Rating Scale, Part III—Motor examination; FT_N1_—Finger-tapping score provided by the first neurologist; FT_N2_—Finger-tapping score provided by the second neurologist; Less AH—The less-affected hand, More AH—The more-affected hand.

**Table 2:** Descriptive statistics (average ± st.deviation) for each feature and group of subjects.

Group	$f_{av}^{\left(i\right)}\;\left(Hz\right)$	$\alpha_{av}\;\left({}^{\circ}\right)$	$i_{dec\;}\left(\#\right)$	$H_{num\;}\left(\#\right)$	$F_{num\;}\left(\#\right)$
**PD**	2.04 ± 0.87	63.08 ± 8.54	5.00 ± 5.66	0–4	0
**MSA**	1.71 ± 1.26	56.27 ± 36.11	4.03 ± 4.74	0–7	0–2
**PSP**	2.37 ± 1.11	44.87 ± 31.74	5.62 ± 4.88	0–4	0–1
**HC**	3.32 ± 0.89	80.48 ± 26.55	11.00 ± 10.99	/	/

**Table 3:** Results of the decision support system for each group of patients separately and in total. The result is provided for two cases: when all recordings are included in the analysis (Case I) and when only recordings with the same score from both raters are included in the analysis (Case II).

Group	Case I Accuracy (%)	Case II Accuracy (%)
**PD**	82.69 ± 2.72	84.00
**MSA**	82.36 ± 8.32	89.65
**PSP**	83.76 ± 7.86	90.91
**TOTAL**	83.33 ± 6.50	88.16

**^1^** PD—Parkinson’s disease; MSA—Multiple system atrophy; PSP—Progressive supranuclear palsy.
